# Cysteine as a Multifaceted Player in Kidney, the *Cysteine-Related Thiolome* and Its Implications for Precision Medicine

**DOI:** 10.3390/molecules27041416

**Published:** 2022-02-19

**Authors:** Maria João Correia, António B. Pimpão, Dalila G. F. Fernandes, Judit Morello, Catarina O. Sequeira, Joaquim Calado, Alexandra M. M. Antunes, Manuel S. Almeida, Patrícia Branco, Emília C. Monteiro, João B. Vicente, Jacinta Serpa, Sofia A. Pereira

**Affiliations:** 1 CEDOC, NOVA Medical School, Universidade Nova de Lisboa, 1169-056 Lisboa, Portugal; mjoao.correia@nms.unl.pt (M.J.C.); antonio.pimpao@nms.unl.pt (A.B.P.); judit.morello@nms.unl.pt (J.M.); catarina.sequeira@nms.unl.pt (C.O.S.); almeidams@sapo.pt (M.S.A.); mateus.meg@gmail.com (P.B.); emilia.monteiro@nms.unl.pt (E.C.M.); jacinta.serpa@nms.unl.pt (J.S.); 2 Instituto de Tecnologia Química e Biológica António Xavier (ITQB NOVA), 2780-157 Oeiras, Portugal; dalilagfhf@itqb.unl.pt (D.G.F.F.); jvicente@itqb.unl.pt (J.B.V.); 3 Centre for Toxicogenomics and Human Health (ToxOmics), Genetics, Oncology and Human Toxicology, Nova Medical School/Faculdade de Ciências Médicas, Universidade Nova de Lisboa, 1169-056 Lisboa, Portugal; jcalado@nms.unl.pt; 4 Nephrology Department, Centro Hospitalar Universitário de Lisboa Central, 1069-166 Lisboa, Portugal; 5 Centro de Química Estrutural, Institute of Molecular Sciences, Instituto Superior Técnico, 1049-001 Lisboa, Portugal; alexandra.antunes@tecnico.ulisboa.pt; 6 Hospital de Santa Cruz, Centro Hospitalar de Lisboa Ocidental, 2790-134 Carnaxide, Portugal; 7 Instituto Português de Oncologia de Lisboa Francisco Gentil (IPOLFG), 1099-023 Lisboa, Portugal

**Keywords:** *cysteine-related thiolome*, cysteine transporters, hypoxia, hypertension, kidney metabolism, glutathione, H_2_S, bioenergetics, ferroptosis, lysosomes

## Abstract

In this review encouraged by original data, we first provided in vivo evidence that the kidney, comparative to the liver or brain, is an organ particularly rich in cysteine. In the kidney, the total availability of cysteine was higher in cortex tissue than in the medulla and distributed in free reduced, free oxidized and protein-bound fractions (in descending order). Next, we provided a comprehensive integrated review on the evidence that supports the reliance on cysteine of the kidney beyond cysteine antioxidant properties, highlighting the relevance of cysteine and its renal metabolism in the control of cysteine excess in the body as a pivotal source of metabolites to kidney biomass and bioenergetics and a promoter of adaptive responses to stressors. This view might translate into novel perspectives on the mechanisms of kidney function and blood pressure regulation and on clinical implications of the *cysteine-related thiolome* as a tool in precision medicine.

## 1. Introduction

Cysteine is a conditional essential amino acid that is mainly obtained through the diet, either directly (meat, fish, whole-grain, soybeans and vegetables) [1] or by de novo synthesis from methionine [2,3,4] (detailed below).

Cysteine is required for protein synthesis, stabilization of the protein structure and one-carbon metabolism and therefore essential for healthy growth and development [5,6]. Cysteine is also a precursor for the synthesis of glutathione, one of the main controllers of redox homeostasis. In addition, cysteine is a source of relevant intracellular metabolites and signaling molecules, including taurine, coenzyme A (CoA), hydrogen sulfide (H_2_S) and cysteine persulfide (CysSSH) [7], all of them playing an essential role in kidney bioenergetics, function and blood pressure regulation (reviewed in References [8,9]).

Similar to other aminothiols, the total cysteine availability in fluids and tissues is a sum of three fractions: a free reduced (CysSH) and two oxidized corresponding to the free oxidized (CysSSX, cysteine disulfides, mainly cystine) and the protein-bound (CysSSP by *S*-cysteinylation of cysteine residues) fractions [10,11]. Cysteine is mostly present in extracellular fluids, wherein it is predominantly found in its oxidized forms [12,13,14].

**Figure 1:**
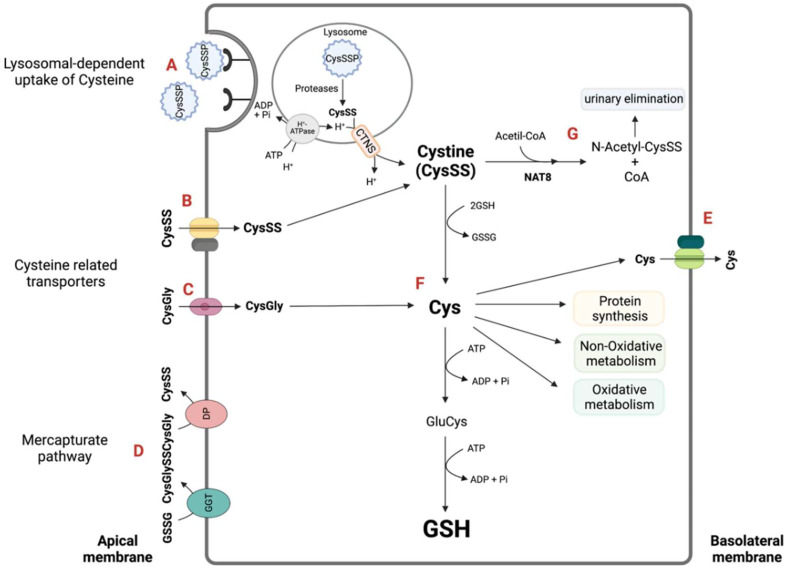
**Cysteine supply and metabolic circuits for the renal epithelial proximal tubular cells.** The net intracellular supply of cysteine is a sum of different contributions. After glomerular filtration, there is an increase in the proximal tubule luminal content of CysSSP and also of glutathione (can also be secreted) and cysteine disulfides (CysSSX, mainly cystine). CysSSP undergoes reabsorption by lysosomal-mediated uptake [30,31,32,33] (**A**). CysSS is reabsorbed by cysteine-related transporters that include the heterodimer b^0,+^AT-rBAT, encoded respectively by SLC7A9-SLC3A1 [34,35]) (**B**). PEPT2 (encoded by Slc15a2) apical influx of CysGly [36] also contributes toto cysteine intracellular availability (**C**). The extracellular thiol pool that nourishes the kidney tubular cell with Cys also has the contribution of the mercapturate pathway (**D**). The GGT, the first enzyme of the mercapturate pathway, has the highest activity in kidney epithelial tubular cells and hydrolyzes glutathione, contributing with CysGly and cysteine disulfides for the pool. The trans-epithelial transport of cysteine involves is the taken up through the brush border membrane (**B**) and its exit through the 4F2hc/LAT-2 transporter at the basolateral membrane [37] (**E**). Once inside the cell, cystine and CysGly are converted in Cys, which may undergo several metabolic circuitries: H_2_S-producing enzymatic pathways, CDO-mediated oxidative metabolism, GSH synthesis and protein incorporation (**F**). Intracellular CysSSX may also be *N*-acetylated by the last mercapturate pathway activity of NAT8 and eliminated in urine (**G**). CDO: cysteine dioxygenase; CTNS: cystinosin; Cys: cysteine; CysGly: cysteinylglycine; CysSSP: cysteinylated proteins; DP: Dipeptidases; GGT: γ-glutamyl transpeptidase; GSH: glutathione; H_2_S: hydrogen sulfide; PTCs: proximal tubular cells. Created with Biorender.com; accessed on 22 January 2022.

CysSSP accounts for approximately 65% of the total plasma cysteine and represents a post-translational modification that prevents the irreversible oxidation of proteins cysteine residues by electrophilic species [13]. CysSSP is a lysosomal-dependent source of cysteine in the kidney epithelial tubular cells [15], together with cysteine provided by the glutathione catabolism through the mercapturate pathway (MAP) [16,17] (detailed below) (Figure 1).

Cysteine is classically described as the limiting precursor of glutathione, which owes its reducing function to the sulfhydryl group (-SH) of cysteine. Glutathione synthesis occurs intracellularly, mainly in hepatocytes, wherein glutathione is in its reduced form (GSH). This cysteine storage in the form of GSH represents a protective mechanism against the toxic effects of cysteine excess [18,19,20].

The fact that an excess of cysteine can result in a net decrease of glutathione levels [21,22] counters the assumption that kidney reliance on cysteine is only related to its channeling for glutathione synthesis. Cysteine can be toxic through its autoxidation [21,22,23] or through the overstimulation of other cysteine metabolic pathways. Despite cysteine storage as glutathione, other perspectives have been dedicated to postulate pheomelanogenesis as an alternative to channel the excess of cysteine [24]. In addition to diet, thermal stress [25,26], infection [27], pollution and other contaminants [28], (epi)genetic determinants of cysteine-related metabolic enzymes [29] may account for individual’s differences in the availability and dynamics of cysteine in the kidney. Likewise, the impairment of the kidney function (e.g., decreased glomerular filtration) or other factors that minimize cysteine consumption/excretion by the kidney might lead to increased circulating CysSSX (e.g., cystine) (detailed in the next sections).

Several particularities of the kidney epithelial tubular cells highlight the relevance of cysteine to the kidney (Figure 1).

MAP is a hallmark of the kidney proximal tubular cells and is relevant for two reasons [16,17,38,39].

First, MAP have an important role as a cysteine supplier (Figure 1D) upon the activities of its extracellular components (γ-glutamyl transpeptidase (GGT) and aminopeptidase) [40,41]. Unlike most cells that are unable to catabolize glutathione or glutathione-*S*-conjugates [42,43], the renal epithelial tubular cells have the highest expression of GGT in the extracellular border of the cell membrane, allowing an efficient recycling of glutathione [20,44]. Glutathione reaches the tubular lumen upon tubular secretion and upon the efflux of oxidized glutathione formed by tubular cells [15]. The impairment of glutathione recycling into cysteine leads to a relevant cysteine deficiency [42].

Second, MAP is relevant as a protector of the renal tubular cell against cysteine-*S*-conjugated toxicity upon the activity of its intracellular component N-acetyltransferase type 8 (NAT8) enzyme (Figure 1G). NAT8 is almost exclusively expressed in the proximal tubular cells of the kidney cortex [45,46]. Cysteine is less likely than glutathione to remain reduced in an oxidative environment [47,48], and the formation of cysteine-*S* conjugates has been reported to be far more stable [49] and with higher half-lives than their precursors [50]. NAT8 is specialized in the control of cysteine-*S* conjugates, including CysSS and other cysteine disulfides [39,46,51], which are acetylated by NAT8 at the expense of acetyl coenzyme A (acetyl-CoA), rendering them prone to urinary elimination (Figure 1G). Importantly, polymorphisms in the NAT8 promotor are associated with systolic blood pressure and chronic kidney disease in hypertensive individuals [45,52,53].

The kidney reliance on cysteine is also demonstrated by the abundance of cysteine-related transporters in the apical membrane of renal epithelial proximal tubular cells [15,34,35,54] (Figure 1B).

The role of cysteine and cystine transporters in renal cysteine circuitries is highlighted by two human mendelian recessive disorders, Cystinuria and Cystinosis, which are caused by mutations in the transmembrane amino acid transport system b^0,+^ and in the lysosomal H^+^-driven cystine efflux (CTNS), respectively (reviewed in Reference [55]).

Cystinuria is characterized by the impaired epithelial transport of cystine and dibasic amino acids (lysine, ornithine and arginine) in the renal epithelial of the proximal tubule (Figure 1B), as well the gastrointestinal tract. This leads to the urinary over-elimination of cystine, which, coupled with its low solubility, causes urolithiases [56]. Mutations in the rBAT heavy subunit coding gene (*SLC3A1*) or the b^0,+^AT light subunit coding gene (*SLC7A9*) underlie Cystinuria, the most common inherited form of kidney stones, with a worldwide prevalence of 1:7000.

Cystinosis, a far rarer disease than Cystinuria, has an incidence of 1:200,000 live births and is a lysosomal transport disorder characterized by the intra-lysosomal accumulation of cystine. The gene responsible, *CTNS*, was positionally cloned in 1998 and found to encode a lysosomal membrane protein, the cystinosin [57]. Cystinosin is a cystine/H^+^ symporter and is therefore H^+^-driven. In addition, it does not transport other amino acids, including the monosulfide cysteine, as the apical rBAT/b^0,+^AT system does, because it is highly specific for L-cystine [55].

Another particularity of kidney proximal epithelial tubular cells, similarly to fibroblasts and leukocytes, is the capability to sustain themselves on cysteine by internalizing extracellular cysteinylated proteins, CysSSP, into lysosomes [30,31,32,33] (Figure 1A). As high levels of cysteine might be toxic, for instance, by inducing protein misfolding and endoplasmic reticulum (ER) stress [58,59,60], this might also constitute a safe mechanism to store cysteine.

The liver–kidney axis accounts for the total cysteine availability. It is interesting to mention that, as shown in rodents, the kidney cysteine-to-glutathione ratio increases up to four times from newborn to adult animals [61], as opposed to a two- to three-fold decrease in the liver. This may reflect the maturation of the liver to provide cysteine and/or the maturation of the kidney to retain and/or use it [42]. Additionally, plasma CysSSP increases with age [13] and in kidney disease [62,63], which may be related to a lower capability of the aging kidney to uptake cysteine.

Increased cystine uptake may render cells highly resistant to oxidative stress, which is particularly relevant upon glutathione depletion [64]. Interestingly, in the case of glutathione depletion, the increase of intracellular cystine does not necessarily affect the cellular glutathione pool. Instead, increased cystine uptake creates a reducing extracellular environment (increased extracellular cysteine) through an efficient Cys/CysSS (cysteine-to-cystine ratio) redox cycle, which may have an important role in cell survival (see Section 3.5).

The cysteine redox equilibrium also contributes to adaptive responses to physiologic and non-physiologic stimuli [10,11,65]. For instance, the redox equilibrium of Cys/CysSS is crucial for endothelial cells [11,66] and the control of vascular health [67], as cysteine and cystine elicit different responses [11,65]. This redox pair equilibrium changes in the kidney upon hypertensive stimuli [10,11] and is independent of the GSH/GSSG ratio. Cysteine redox cycling has important consequences in DNA repair, cell proliferation, apoptosis, ER stress and inflammatory response [68], the functional inactivation of enzymes, activation of the unfolded protein response [69] and antioxidant response elements [23,70,71]. This may also imply that kidney-related diseases, such as hypertension (HTN), might be related to the disruption of cysteine redox signaling/equilibrium [65].

The study of the redox and detoxification activity of thiols have been mainly dedicated to glutathione in liver, and cysteine has been classically described as its precursor. While lesser information exists for the kidney, the fact that glutathione synthesis from cysteine has two ATP-requiring steps (Figure 1) [20] may elicit that, in order to fulfill the kidney’s high energy demand, the abundance of cysteine in the kidney might contribute to other purposes than the classical GSH ones.

Moreover, the dynamics of thiols between their fractions need better characterization, including its organ dependence in health and disease. Most studies on glutathione investigate the ratio GSH/GSSG and in what comes up for cysteine, the terminology Cys is mentioned not allowing to discriminate free from oxidized forms, and sometimes, even cyst(e)ine is stated, while it is well-known that cysteine and cystine have different biologic effects.

While being cautious on the species differences that may occur, the study of the mechanisms that regulate the amount of cysteine that reaches the kidney and that re-mains in the body requires a systems biology approach that cannot be achieved in cellular models. In fact, we provided evidence that there is a change in cysteine dynamics in kidney of animal models of HTN that is dependent on the hypertensive stimuli used, i.e., chronic intermittent hypoxia [10,11] vs. high salt consumption [72]. We also observed that cysteine, glutathione and cysteinylglycine have independent dynamics between its three different fractions [10,11,72]. We defined the net organ variation of these thiols dynamics as *cysteine-related thiolome*. Herein, in Section 2, we present original data of the *cysteine-related thiolome* for the liver, the kidney cortex and kidney medulla and brain hippocampus and cortex tissue in a healthy Wistar rat.

## 2. Results

### 2.1. Cysteine-Related Thiolome in Different Organs

A set of experiments was designed in order to get a general view of *cysteine-related thiolome* in different tissues (liver, kidney and brain) of healthy Wistar rats. Study design and methodologies are described in Section 5 at the end of the manuscript.

**Figure 2:**
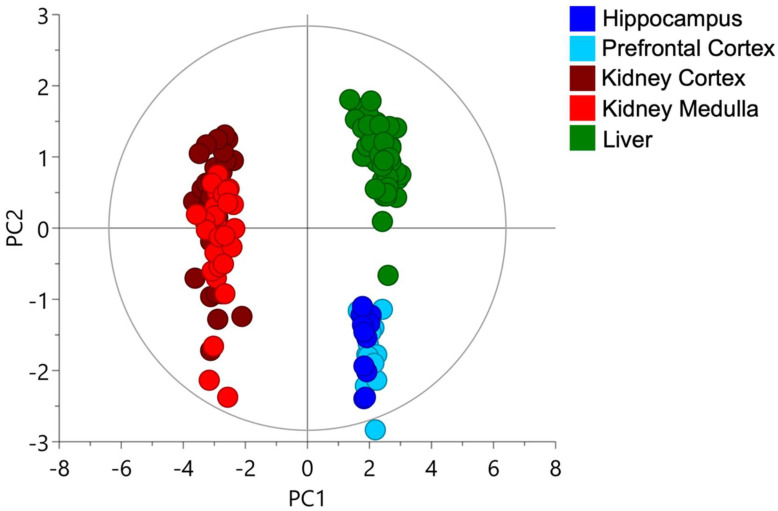
Organ dependence of the *cysteine-related thiolome*. Principal Component Analysis (Score plot) with all studied organs; first two components covered 77 and 15% of the variance of the data, respectively. A total of 17 samples of brain tissue, 33 of kidney tissue and 53 of liver tissue were analyzed. PC: Principal Component.

A Principal Component Analysis (PCA) was performed with all the different fractions of glutathione, cysteine and cysteinylglycine that were quantified for all the studied tissues. The PCA is a multivariate analysis commonly used to assess the main sources in the variance of the data. We found that the two first components explained 77 and 15% of the variance of the data, respectively. The plot score showed a clear separation between kidney, brain and liver tissues (Figure 2) with a distinct *cysteine-related thiolome* of the kidney when compared with the liver or the brain. We could not differentiate hippocampus from prefrontal cortex in the brain, although the kidney cortex had marginally higher cysteine levels when compared to the medulla.

### 2.2. The Kidney Is a Cysteine-Rich Organ

**Figure 3:**
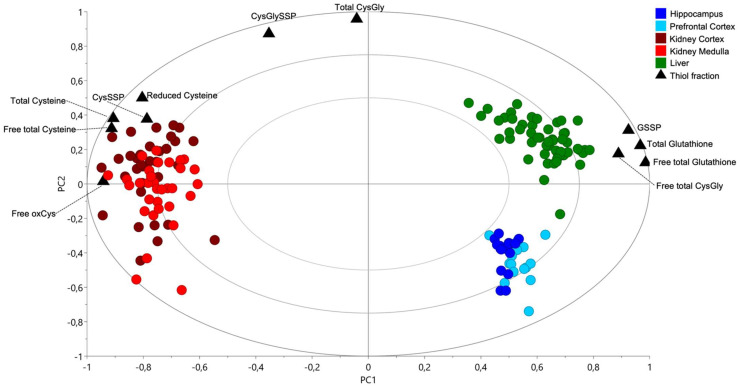
Tissue-specific thiol distribution amongst the organs. Principal Component Analysis with all organs; biplot displaying the plot score and the loadings plot. CysGly: cysteinylglycine; CysGlySSP: cysteinylglycinated proteins; CysSSP: cysteinylated proteins; GSSP: glutathionylated proteins; oxCys: oxidized cysteine; PC: Principal Component.

A biplot, which overlies the plot score, and loadings plot of a PCA were built to identify the most relevant thiol fractions for each organ. The kidney presented higher concentrations of all cysteine fractions than the liver or the brain (Figure 3). The liver was richer in total, free total and protein-bound glutathione and in free total cysteinylglycine than the kidney (cortex or medulla) or the brain (hippocampus or prefrontal cortex) (Figure 3).

**Figure 4:**
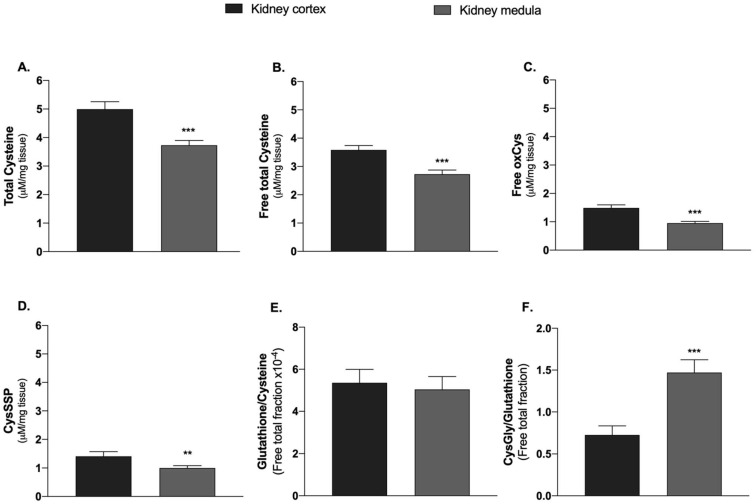
Cysteine-related thiols in the kidney cortex and kidney medulla. (**A**) Total cysteine. (**B**) Free total cysteine. (**C**) Free oxidized cysteine. (**D**) Cysteinylated protein. (**E**) Free total glutathione/Free total cysteine (Glutathione synthesis). (**F**) Free total cysteinylglycine/Free total glutathione (Glutathione catabolism). Data are presented as the mean ± standard error of the mean. Wilcoxon matched-pairs signed rank test, ** *p* < 0.01 and *** *p* < 0.001. CysGly: cysteinylglycine; CysSSP: cysteinylated proteins; oxCys: oxidized cysteine.

Univariate analysis of the kidney thiol levels demonstrated that the kidney cortex had higher levels of all cysteine fractions than kidney medulla (Figure 4A–D). In these tissues, the most abundant fraction was the reduced cysteine (2.09 ± 0.16 and 1.77 ± 0.13 μM/mg of tissue for the cortex and medulla, respectively) (data not shown), followed by the free oxidized and protein-bound fractions (Figure 4C,D). While the indicator of glutathione synthesis (free total glutathione-to-free total cysteine ratio) was similar in both renal tissues (Figure 4E), the indicator of glutathione catabolism (free total cysteinylglycine-to-free total glutathione ratio) was higher in the kidney medulla (Figure 4F).

The kidney richness in cysteine compared to the other organs lead us to think that cysteine is highly relevant for the kidney that might rely on it to properly function and that this high cysteine content may have an adaptive purpose. In the next section, we review the evidence that may support our understanding of what might justify the kidney’s reliance on cysteine.

## 3. Evidence Supporting That Cysteine and Its Renal Metabolism Justify the Kidney’s Reliance on Cysteine: A Literature Review

Since excess cysteine is toxic, its metabolism is crucial to keep cysteine levels below the threshold of toxicity [73]. The total cysteine availability in the kidney of each individual will reflect the contribution of the cysteine supply and, also, the individual metabolic capacity [74].

### 3.1. Cysteine De Novo Synthesis

Cysteine can be synthesized de novo from dietary methionine through the reverse trans-sulfuration pathway (RTP) that branches from the methionine demethylation/remethylation cycle. Under certain physiological conditions, homocysteine derived from *S*-adenosylhomocysteine hydrolase is condensed with serine through the action of cystathionine β-synthase (CBS) to yield cystathionine, which is then converted to cysteine (and α-ketobutyrate and ammonia) by cystathionine γ-lyase (CSE). Both pyridoxal 5′-phosphate (PLP)-dependent enzymes are significantly expressed in kidney tissues, and the RTP has been shown to be fully functional in the kidney [75,76], thereby acting as a putative contributor for the cysteine pool in kidney [2,3,4]. Methionine is present at up to 10-fold higher intracellular concentrations than cysteine [77]. Kidney injury downregulates the expression of the RTP enzymes in the kidney, leading to increased oxidative stress and inflammatory response [78], justifying cysteine abundance as a protective factor. Notably, besides functioning as a cysteine source, the RTP enzymes are responsible for the clearance of homocysteine, affording its deviation from the methionine demethylation/remethylation and thereby preventing its accumulation into toxic levels. Indeed, excess plasmatic homocysteine, designated by (hyper)homocyst(e)inemia, has been posited as an etiological agent for renal and cardiovascular [79,80] disease, although the pathogenic mechanisms remain to be elucidated.

### 3.2. Cysteine Catabolism in the Control of Cysteine Excess and a Source of Relevant Metabolites

There are different pathways for cysteine catabolism that produce a variety of metabolites with active (patho)physiological roles.

In cysteine oxidative catabolism, cysteine is converted to cysteine sulfinic acid (CSA) through cysteine dioxygenase (CDO) enzyme (an irreversible reaction), using oxygen as co-substrate. CSA can be further metabolized in two routes [81]: the most frequent is the conversion of CSA to pyruvate and sulfate by a transamination pathway, and the least frequent is the conversion of CSA by the cysteine sulfinate decarboxylase (CSD) into hypotaurine, which is further oxidized to taurine [81]. CDO competes with γ-glutamylcysteine synthetase (GCS), as both use cysteine as substrate and are mainly located in the liver [73,81,82]. CDO is also detected in the kidney and, to a much lesser extent, in the brain of rodents [81], but its renal expression in humans is largely unknown. CDO’s main physiological action is the prevention of cysteine excess and to form taurine and pyruvate precursors. However, in humans, due to low CSD activity, endogenous taurine synthesis is very limited [83,84,85]. Moreover, cysteine might originate CoA (through the pantothenate pathway) [86], which can be degraded into cysteamine, whose only known function is to be a precursor for the formation of hypotaurine, by cysteamine dioxygenase [87], which is further oxidized to taurine. Thus, while a better knowledge on these thiol dioxygenases is needed, and although the exogenous administration of taurine and cysteamine have beneficial effects at the cardiovascular and renal levels [88,89,90,91,92,93,94,95], the endogenous synthesis of taurine might not represent a major factor for the kidney reliance on cysteine.

One of the major cysteine catabolic pathways with greater relevance for kidney and cardiovascular function consists of the production of the secondary messenger H_2_S by the RTP enzymes CBS and CSE and 3-mercaptopyruvate sulfurtransferase (MST, coupled with cysteine aminotransferase, CAT). While CBS and CSE were historically considered for their ‘canonical’ activity in terms of de novo cysteine synthesis, their catalytic versatility enables CBS and CSE to act as major enzymatic sources of H_2_S, a reactive molecule of gaseous nature that is endogenously produced to accomplish signaling functions. CSE expression is higher than CBS in both kidney and liver. CBS is threefold more abundant in the kidney than in the liver [96]. CBS is thus possibly relevant for H_2_S generation in the kidney [97]. H_2_S modifies target proteins affecting their function, structure and stability by two distinct mechanisms: binding to and/or interacting with transition metal cofactors or by modulating the persulfidation of cysteine residues, a post-translational modification that results in the addition of sulfane sulfur to a cysteine sulfhydryl moiety (detailed below). In general, protein persulfidation affords protection from damaging oxidative modifications to the respective cysteine residues [98], which have been shown to provide protection in acute kidney injury (reviewed in Reference [76]). Moreover, in different models, aging has been associated with decreasing protein cysteine persulfidation (CysSSH) concurrent with increasing protein cysteine sulfenylation (CysSOH) [99]. A recent study on the effect of dietary restriction in a mouse model revealed 1086 persulfidated kidney proteins, 16 of which become enriched under such dietary conditions with respect to ad libitum-fed mice [100], attesting the prevalence of this modification and its adaptive nature.

The ability of CBS and CSE to synthesize H_2_S involves different combinations of substrates, the most effective consisting of the β-replacement of cysteine and homocysteine into cystathionine and H_2_S (reviewed, e.g., in Reference [101]). Notably, both enzymes can also catalyze the β-replacement of two cysteine molecules, yielding H_2_S and lanthionine, which have been posited as uremic toxins associated with vascular calcification in chronic kidney disease [102,103]. In addition to CBS and CSE, CAT [104] deaminates cysteine into 3-mercaptopyruvate, which is used as a substrate of MST to yield H_2_S and pyruvate. While all three H_2_S-synthesizing enzymes can be localized to kidney cells, their increased expression in renal cancer cells (both clear cell and non-clear cell carcinoma) with respect to adjacent normal tissues has been reported (reviewed, e.g., in Reference [105]). Recently, along with H_2_S-synthesizing enzymes, the selenium-binding protein 1 (SELENBP1) has also been shown to generate H_2_S [106]. Notably, this enzyme is considered a marker of kidney injury and metal-induced nephrotoxicity (e.g., in Reference [107]). Local production of H_2_S within the kidney has been reported as a promoter of glomerular filtration and inhibitor of tubular sodium reabsorption; therefore, it is a stimulator for natriuresis and diuresis [108]. After the tubular cell’s injury, a process of fibrosis occurs. Even in this scenario, the crucial role of cysteine catabolism is evident in the normal functioning of renal epithelial tubular cells, since the metabolic players CBS and CSE are downregulated upon fibrosis, as well as the levels of H_2_S [109].

H_2_S homeostatic levels are maintained through a fine balance between its synthesis and consumption by a mitochondrial sulfide oxidation pathway (mSOP), comprising four enzymes. H_2_S is initially oxidized by sulfide:quinone oxidoreductase (SQR), which transfers a sulfane sulfur to GSH, yielding glutathione persulfide (GSSH) while reducing ubiquinone to ubiquinol. In this manner, H_2_S metabolism directly links cysteine catabolism with mitochondrial bioenergetics (detailed below). GSSH is then used by persulfide dioxygenase to generate sulfite and GSH. Subsequently, thiosulfate sulfurtransferase (TST, or rhodanese) converts GSSH and sulfite into thiosulfate. Sulfite can also be oxidized to sulfate by sulfite oxidase. The final products of cysteine-derived H_2_S oxidation are thus thiosulfate and sulfate. A nonfunctional mSOP due to ubiquinone deficiency in mouse kidney results in sulfide accumulation, increased oxidative stress, inhibited fatty acid oxidation and kidney failure [110]. Notably, in a mouse model depleted of TST, an adaptive hepatic response to increased systemic sulfide levels has been shown to promote diabetes [111].

Cysteine can also be employed to generate free cysteine persulfide (CysSSH), a low molecular weight signaling molecule with an additional sulfane sulfur moiety. CysSSH has increased nucleophilicity and electrophilicity with respect to its thiol counterpart, thereby enabling its prompt reaction with cysteine residues in target proteins. Two main routes have been reported to generate CysSSH. The major enzyme involved in CysSSH production from cysteine in physiological conditions has been proposed to be the mitochondrial cysteinyl-tRNA synthetase (CARS2) [112]. Besides synthesizing CysSSH, CARS2 has been proposed to co-translationally insert CysSSH into nascent polypeptides, thereby generating proteins that are persulfidated a priori. Under pathophysiological oxidative conditions such as those encountered in kidney cells in chronic kidney disease, an important role has been assigned to CBS and CSE in CysSSH production from cystine [113]. The intracellular accumulation of cystine can thus generate increased production of CysSSH, which is expected to affect global protein persulfidation.

### 3.3. Cysteine Is a Pivotal Molecule for Kidney Biomass and Bioenergetics

**Figure 5:**
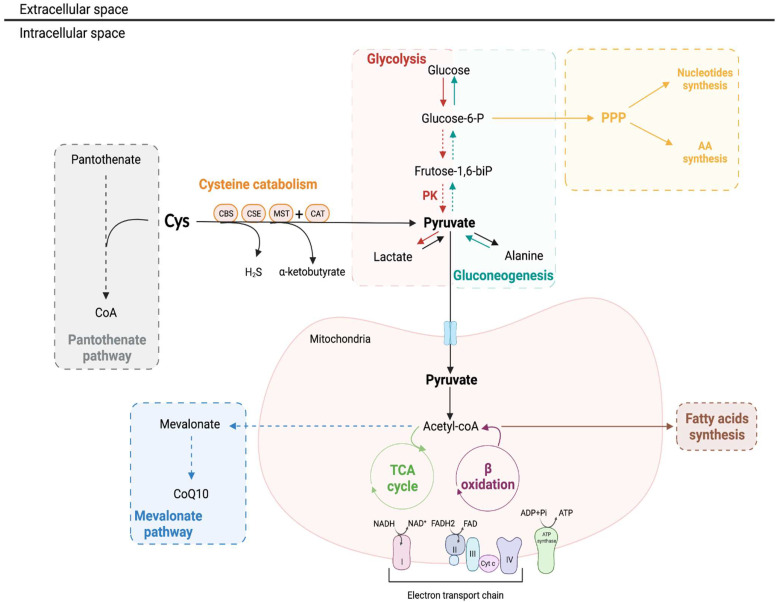
Cysteine metabolism as a source for biomass and bioenergetics. Cys catabolism generates several intermediates that are involved in several metabolic pathways. Through the action of CSE or MST and CAT, besides generating H_2_S, Cys generates pyruvate, which can supply the TCA cycle and consequently contribute to energy production. Pyruvate can also be converted into alanine and lactate. The products of Cys metabolism can also be deviated to the PPP, which is involved in both nucleotide and amino acid synthesis. In the kidney, the main source of energy is fatty acid β-oxidation, and since the pyruvate resulting from Cys catabolism can also originate acetyl-coA, this suggest Cys as an alternative acetyl-CoA supplier for oxidative phosphorylation and fatty acid synthesis. AA: amino acid; CAT: cysteine aminotransferase; CBS: cystathionine β-synthase; CoA: coenzyme A; CoQ10: coenzyme Q10; CSE: cystathionine γ-lyase; Cys: cysteine; Cyt c: cytochrome c; H_2_S: hydrogen sulfide; MST: 3-mercapto-pyruvate sulfurtransferase; PPP: pentose phosphate pathway; TCA: tricarboxylic acid. Created with Biorender.com; accessed on 22 January 2022.

The contribution of cysteine as an energy and biomass source remains to be thoroughly investigated in the kidney, but it represents a biological possibility based on the metabolic circuitries described for other high ATP-demanding cell models [114]. For instance, it is useful as a carbon source (i.e., glucose, fatty acids and amino acids) with a significant intervention in biosynthesis and bioenergetics (as an alternative supplier of metabolites for β-oxidation and tricarboxylic acid cycle, TCA, and electron equivalents for the mitochondrial electron transport chain, mETC) [7,114,115,116,117,118,119] (Figure 5).

Cysteine may be part of the adaptation of energy metabolism to sustain mitochondrial ATP production, partially underlying the kidney reliance on cysteine. Cysteine promotes the adaptation of cells to metabolically damaging conditions, and it can also be consumed at different spots of the metabolic network.

#### 3.3.1. β-Oxidation and Oxidative Phosphorylation

The proximal tubule consumes high levels of ATP to accomplish solute and nutrients reabsorption and to maintain the electrolyte balance. As previously mentioned, mitochondrial fatty acid β-oxidation serves as the preferred source of ATP by the kidney [120]. Accordingly, proximal tubular cells contain more mitochondria than other renal cell types [121]. Both ATP depletion and lipotoxicity may elicit tubular injury and fibrosis progression (reviewed in Reference [122]). Fatty acid β-oxidation is a primordial energetic process to fulfill the kidney energetic requirements and wherein broken-down fatty acids are a fuel for bioenergetics. As a precursor of CoA via the pantothenate pathway, cysteine can also influence fatty acid β-oxidation. CoA is added to the free fatty acids in the early steps of β-oxidation by acyl-CoA synthetase, allowing its activation in fatty acyl-CoA and posterior incorporation into the outer mitochondrial membrane (reviewed in Reference [123]). Once in the mitochondrial matrix, the fatty acyl-CoA undergoes β-oxidation, producing one molecule of acetyl-CoA in each cycle. The resulting acetyl-CoA then enters the TCA cycle. Both β-oxidation and the TCA cycle produce NADH and FADH2 to be used by the mETC to generate ATP. The excess acetyl-CoA can then be exported outside the mitochondria and be used for the synthesis of new fatty acids (reviewed in Reference [124]). The impairment of fatty acid β-oxidation is related to a reduced ATP production, a condition that is common in several nephropathies (e.g., acute kidney injury, diabetic nephropathy and chronic kidney disease) [121,125].

Due to their low glycolytic activity, kidney proximal tubular cells rely on mitochondrial oxidative phosphorylation to provide for ATP, using mostly non-esterified free fatty acids (primarily palmitate) and, to a lesser degree, lactate, citrate and pyruvate as preferential energy substrates [126]. Other substrates may include glutamine and ketone bodies. We found that the cysteine levels are higher in the kidney cortex than in kidney medulla (Figure 4A). For instance, the renal cortex uses oxidative phosphorylation fed mainly by fatty acid β-oxidation of fatty acids and low amounts of glucose, while the renal medulla uses anaerobic glycolysis due to low oxygen levels [121,127]. Cysteine might be a source of both pyruvate and lactate, which are an alternative source for oxidative phosphorylation and, consequently, for ATP production.

Amongst the core glucose-related pathways, gluconeogenesis and phosphate pentose pathway (PPP) are prominently placed in the kidney (Figure 5).

#### 3.3.2. Gluconeogenesis

Gluconeogenesis is the de novo synthesis of glucose from non-glucidic compounds, and it is a reversion of glycolysis, with three alternative reactions overcoming the irreversible steps of glycolysis [128,129]. Cysteine is a gluconeogenic amino acid, as it gives rise to pyruvate. Despite its low glycolytic capacity, the proximal tubule is the sole segment of the nephron with appropriate enzymes for gluconeogenesis [130,131,132,133,134], competing with the Na^+^/K^+^ ATPase pump for ATP. For that reason, renal epithelial proximal tubular cells have a higher demand of ATP [135]. Additionally, cysteine has been pointed out as an important regulator of enzymes, such as peroxidases that can interact with the pyruvate kinase and block the synthesis of acetyl-CoA from pyruvate, preventing pyruvate entrance in the TCA cycle or in fatty acids synthesis [136] and favoring its deviation into gluconeogenesis, guaranteeing the cell demands for glucose.

The main gluconeogenic amino acids are glutamine and alanine, and considering the contribution of hepatic and renal gluconeogenesis for the systemic glucose pool, it was described that the kidney predominantly contributes to glutamine-derived glucose, whereas the liver contributes to alanine-derived glucose [137]. However, small amounts of alanine-derived glucose come from the renal gluconeogenesis [137], and alanine can be a cysteine-derived amino acid [138]. Moreover, the kidney might be unique in its capacity to release some of the produced pyruvate back into the circulation, thus supporting renal cysteine as a contributor to whole-body energy homeostasis [139].

Gluconeogenesis is a kidney intrinsic metabolic pathway, and it produces higher amounts of glucose than hepatic gluconeogenesis in a gram-for-gram comparison [140,141]. In pathological conditions, such as obesity and diabetes, gluconeogenesis is increased in renal cells [140,142], and its real contribution for the systemic yield of glucose and insulin resistance remains under study [143]. Interestingly, studies in cultured human proximal tubular cells showed that the insulin-induced reactive oxygen species (ROS) production was responsible for the upregulation of sodium-glucose cotransporter-2 (SGLT2, encoded by SLC5A2 gene) and increased glucose uptake [144]. The latter can be inhibited by *N*-acetylcysteine (NAC) administration, supporting a link between glucose metabolism and cysteine that needs further clarification.

#### 3.3.3. Pentose Phosphate Pathway

The inhibition of the final step of gluconeogenesis channels glucose-6-phosphate to the pentose phosphate pathway (PPP), making gluconeogenesis a supplier of PPP in the context of glucose scarcity. Again, cysteine, as a source of pyruvate that is a substrate for gluconeogenesis and is subsequently a PPP supplier. The PPP presents two irreversible oxidative reactions followed by two biochemical branches (oxidative and nonoxidative) of reversible reactions, and it occurs in parallel to glycolysis [145]. The nonoxidative branch of PPP uses glucose-6-phosphate to generate pentose phosphates for the synthesis of amino acids and nucleotides. The oxidative branch of PPP is responsible for the generation of NADPH, supporting fatty acid synthesis and contributing for redox balance [146,147,148,149]. Besides its role in whole metabolic network, NADPH is required for the intracellular conversion of cystine to cysteine, making cysteine available for several metabolic directions [150].

In acute kidney injury, the activation of PPP occurs [151], and it may be a way of reinforcing the biosynthetic and redox capacity of injured cells as an attempt of sustaining cell viability and rescuing the organ’s function. In line with this, the increased glycolytic rate was reported as contributing to renal cell integrity and viability in ischemic conditions, in part because glycolysis supplies PPP [152,153]. The energetic demands rely more on the oxidative metabolism, until a certain limit [154], as the increase of oxidative phosphorylation is essential to avoid necrosis in renal proximal tubular cells upon injury [155]. As reported, glycolysis and oxidative phosphorylation can work simultaneously in renal cells, with different contributions for physiological metabolism and for function rescue upon injury, and as mentioned before (Section 3.3.1), cysteine can be a valuable source to sustain oxidative phosphorylation.

Another connection of gluconeogenesis and PPP with cysteine is Nrf2. This transcription factor is a master regulator of redox control and promotes cysteine bioavailability and GSH synthesis, which are pivotal in PPP oxidative branch activity [156,157].

These are consistent clues that non-glucose-derived organic compounds are contributing for the maintenance of oxidative metabolism, keeping the pool of pyruvate high upon the disturbance of glucose catabolism, cysteine certainly being a good candidate.

### 3.4. Cysteine as an Alternative Source of Energy through H_2_S Production

Cysteine can also serve as an alternative source of energy through the production of H_2_S. H_2_S has a double-faced behavior with regard to cell bioenergetics. Regarding mETC, at low levels, H_2_S can sustain cellular respiration by supplying reducing equivalents through SQR, which reduces ubiquinone to ubiquinol that can then donate electrons to complex III (reviewed, e.g., in References [158,159]). However, at higher levels (high nM–low μM), H_2_S becomes inhibitory towards complex IV, binding to its heme-copper active site, blocking oxygen reduction and preventing proton translocation and consequently ATP production by ATP synthase [160]. The potential toxicity of H_2_S and the requirement to dispose of it once it reaches high inhibitory levels for mETC has been recently shown in colonic epithelial cells where a reversal of complex II activity leads to its function in a redox cycle with SQR, thereby detoxifying excess H_2_S and leading to succinate accumulation [161]. In addition to the multiple control points where H_2_S regulates mETC, H_2_S-mediated protein persulfidation also affects key enzymes in cellular bioenergetics. Persulfidation of residues Cys244 and Cys294 in ATP synthase has been shown to stimulate its activity [162]. Moreover, H_2_S has been demonstrated to stimulate glyceraldehyde 3-phosphate dehydrogenase (GAPDH) and the pyruvate-to-lactate converting activity of lactate dehydrogenase (LDH) through persulfidation of specific cysteine residues [163,164], reviewed in Reference [159]. The significant extent of protein persulfidation and its modulatory effect enabling metabolic reprogramming in face of different challenges hints at a strong impact of H_2_S and its precursor cysteine in the adaptation to such challenges.

### 3.5. Cysteine Is Preventive of Ferroptosis and Promoter of Cell Survival

High levels of cysteine import may be an adaptive feature to inhibit ferroptosis in vivo [165,166]. Ferroptosis is a form of regulated cell death that is iron-dependent, caused by an accumulation of lipid peroxidation/ROS [167,168]. Physiologically, this process is prevented by the glutathione peroxidase 4 (GPX4), which uses glutathione as a co-substrate to detoxify lipid peroxides [169,170] to prevent the massive cell death of kidney tubular epithelia [169]. Ferroptosis is activated upon several injuries (reviewed in Reference [171]), including ischemia/reperfusion injury and drug-induced toxicity.

Recent in vitro studies demonstrated that, in order to avoid ferroptosis, cysteine uptake is required [167,172]. Moreover, ferroptosis is triggered in a situation of cystine deprivation [173]. Contrarily to what would be expected, glutathione depletion seems to be insufficient to induce ferroptosis [174]. Under such conditions, GPX4 might employ alternative reducing molecules, like cysteine and its metabolite CoA [165,175]. Glutathione catabolism [176], compared to the RTP [177], may be a more efficient alternative source of cysteine to avoid ferroptosis, at least in short-term cysteine deprivation. The CoA-dependent post-translational modification, named CoAlation [178], of glycolytic enzymes may boost NADPH synthesis via the PPP, affording protection from ferroptosis [179].

Since cysteine can be converted to acetyl-CoA, it also allows the synthesis of coenzyme Q10 (CoQ10) (via the mevalonate pathway) [180], a key metabolite for preventing ferroptotic cell death [175,181], which is also associated with blood pressure control [182]. CoQ10 binds to the lipid peroxyl radicals, and together with the ferroptosis suppressing protein 1 (FSP1), the FSP1/CoQ10/NADPH axis represents a parallel system to the classical GPX4/glutathione to suppress lipid peroxidation and prevent ferroptosis [175,181].

In addition, cysteine can be metabolized by NFS1 cysteine desulfurase to provide sulfur for the assembly of iron–sulfur (Fe–S) clusters, promoting a response to iron starvation and to ferroptosis [183].

The protective role of cysteine can also be mediated through the formation of H_2_S, whose exogenous administration in vitro (as NaHS) has demonstrated a protective role against ferroptosis induced by RSL3 (a ferroptosis agonist) through the inhibition of ALOX12 acetylation and membrane phospholipid oxidation [184].

Thus, since cysteine is involved in protein synthesis and is a necessary precursor for several molecules including glutathione, CoA and Fe–S clusters [114], the loss of intracellular cysteine can induce ferroptosis at least in five different ways. Kidney tubular cell death induced by transforming growth factor-β1 (TGF-β1) is mediated by a reduction of cysteine influx that leads to ferroptosis-induced cell death [185]. Therefore, cysteine must be viewed as a discrete major regulator of cell survival.

### 3.6. Cysteine Contribution for Kidney Adaptation to Hypoxia

Chronic hypoxia is a common event in kidney disease [186,187], including ischemia/reperfusion- or nephrotoxin-induced acute renal failure, the progression of chronic nephropathies, diabetic nephropathy and HTN (reviewed in Reference [108]). The role of cysteine in the adaptation to hypoxic conditions has been recognized in cancer by inducing a metabolic reprogramming of the cells in order to cope with a high metabolic demand and challenging oxidative stress conditions [188]. A deeper knowledge regarding kidney (patho)physiology is necessary.

There are several factors that render the kidney highly susceptible to hypoxia (reviewed in Reference [189]). This includes a high need for ATP and the obligate aerobic metabolizer status of the proximal tubule [190,191]. The oxygen tension (PO_2_), energy metabolism and blood flow vary among kidney regions. For instance, H_2_S oxidation in the mitochondria represents an alternative source for the synthesis of ATP (see Section 3.4), which is dependent on the PO_2_ [192]. The PO_2_ is higher in the renal cortex than in renal medulla [134,193,194,195,196]. In physiological conditions, the majority of H_2_S is rapidly oxidized in the renal cortex [197,198]. The oxygen availability in the kidney medulla differs from that in the kidney cortex, as the renal medulla has a hypoxic environment (PO_2_ = 5–15 mmHg). As so, medullary mitochondria are more adapted to maintaining a high metabolic activity in an oxygen-deprived milieu than renal cortical mitochondria [199]. Given this, it is expected that the activity of H_2_S in the medullary region is higher than in the kidney cortical region [108], which might function as an oxygen sensor to restore O_2_ balance by increasing medullary blood flow, reducing energy requirements for tubular transport and directly inhibiting mitochondrial respiration [108]. Indeed, different mechanisms linking H_2_S metabolism and hypoxia adaptation have been demonstrated. CBS has been shown to be localized in liver mitochondria upon hypoxia/ischemia, affording the possibility to generate H_2_S in situ, this enzyme becoming degraded by the Lon protease upon restoration of O_2_ levels [200]. In colorectal cancer cells, prolonged exposure to hypoxia promoted an enrichment of mitochondria in H_2_S disposal capacity by increased SQR expression [201]. Recently, Marutani et al. demonstrated an inverse correlation between SQR expression and brain sensitivity to hypoxia [202]. Thus, the depletion of endogenous H_2_S may contribute to the pathogenesis of hypoxia-related kidney pathologies [108,203]. In fact, the administration of H_2_S donors has been demonstrated to possess therapeutic properties under hypoxic conditions [108].

Another particularity of the kidney is that its endothelial cells have a reduced proliferative capacity [204], partly due to a hyporeactivity to proangiogenic factors, which might be due to an unknown negative regulatory mechanism [204]. It has been suggested that hypoxia does not interfere with angiogenesis in kidney endothelial cells to prevent the impairment of hematocrit regulatory mechanisms [189], a balance between the erythropoietin stimulation of erythrocyte production and also control of the blood volume by modulating salt and water excretion. Hypoxia elicits renal production of erythropoietin [205,206] and also acutely stimulates diuresis and natriuresis [207,208], promoting hemoconcentration. The exact link between cysteine metabolism and this mechanism remains to be clarified. However, cysteine has been described as an inhibitor of endothelial cells activation [209] and angiogenesis, which may support this evolutionary trade-off of the kidney towards the regulation of the extracellular fluid volume and blood oxygen carrying capacity rather than protection from hypoxia.

In rats exposed to hypobaric hypoxia, the supplementation with NAC, mimicking high cysteine availability, leads to an increase in brain H_2_S levels mediated by CBS [210], which might be upregulated by HIF-1 [211], the master regulator of hypoxic gene expression [212]. The elevated H_2_S levels might also stabilize HIF-1α and its target gene VEGF [213]. In addition, the supplementation of human endothelial cells (HUVECS) with cystine, contrarily to cysteine, promoted an increased expression of HIF-1α [11]. Collectively, this evidence supports that cysteine availability, together with the optimal activity of cysteine metabolic enzymes, contributes to HIF-1α stabilization and might enable the adaptation to hypoxia [210].

Renal hypoxia may occur in HTN [214,215,216,217], but its contribution for the development of HTN is less clear. In an animal model of HTN related to mild obstructive sleep apnea, wherein increased blood pressure was induced by chronic intermittent hypoxia but without fibrosis-related histologic findings, there was a change in *cysteine-related thiolome* in the kidney cortex, namely an increase in the total glutathione availability and in protein cysteinylation and cysteinylglycinylation, which preceded HTN development [11]. The increase of these fractions was not observed in established HTN, wherein the kidney cortex was already under a hypoxic state [11,217]. While the underlying mechanisms are still to be unveiled, this suggests a link between *cysteine-related thiolome* and the adaptive response to hypoxia. This link is also supported by data on the HIF stabilizer enarodustat that prevented GSSG accumulation in the renal tissues of a rat model of type I diabetes [218].

### 3.7. Implications for Precision Medicine in Arterial Hypertension and Kidney Disease

The differences in the way of handling cysteine, its absorption, storage, metabolism and excretion might vary among individuals. The differences between organs in both cysteine storage and usage might imply different strategies to maintain cysteine in healthy concentrations, when the kidney is not able to handle it or, in the opposite direction, when there is deficit of cysteine in the kidney. In this way, using the cysteine metabolic players and its metabolic products might represent a precision medicine tool to stratify and follow individual renal-driven metabolic changes and blood pressure regulation.

For instance, cystine levels are increased in peripheral blood of patients undergoing hemodialysis (HD) [203], which can reinforce, at least in part, the metabolic importance of cysteine in the kidney, not only because the damaged kidney is no longer working properly in the redox control and clearance functions, but also because cells are not consuming cysteine needed for proper function. Additionally, a recent study showed that increased concentrations of cystine were associated with older age, longer HD duration and higher body mass index (BMI), demonstrating that higher cystine levels predicted both cardiovascular-related and all-cause mortality in HD patients [219]. Increased plasma cystine has been associated with aging, decreased estimated glomerular filtration rate, increased BMI, diabetes and HTN [220,221] and increased risk of mortality [220,221,222,223]. A higher prevalence of HTN is also found among patients with cystinuria, particularly in male patients [224].

While more evidence is needed regarding cysteine [10,11,225,226], the renal tubular metabolism has recently emerged as a contributor to the long-term regulation of blood pressure (reviewed in Reference [80]). This metabolic perspective is co-substantiated by the impact on blood pressure that has been attributed to SGLT2 inhibitors [227], HIF stabilizers [228], gut-microbiota driven-metabolites (reviewed in References [227,229,230]) or to genome-wide association studies of environment-related metabolic pathways with high expression in kidney tubule, including the MAP [39] and the aryl-hydrocarbon receptor (AhR) circuitry [11,230,231]. Thus, more clinical and mechanistic studies are needed in interindividual variability in cysteine dynamics that may select patients more vulnerable to increased blood pressure and the *cysteine-related thiolome* might represent a reliable indicator of this vulnerability.

In fact, clinical trials involving dietary intervention for the reduction of salt consumption showed a positive association between urinary cystine and increased blood pressure [79] and that cystine is a urinary metabolite hat predict the classification of blood pressure salt-sensitivity. In Dahl salt-sensitive rat model of HTN, the animals fed with high salt (HS) diet had an increase in CysSSP in the kidney concomitantly with increase blood pressure and kidney damage. When the animals were fed with a HS berry-enriched diet, in addition to beneficial effects in blood pressure and in kidney histologic findings, there was a decrease in CysSSP and in the total cysteine availability in the kidney [72], showing that dietary interventions might impact the *cysteine-related thiolome*.

A note on antioxidant therapies, which aim to target the oxidative stress burden that is a continuum in development of HTN from hemodynamic changes to inflammatory response, vascular dysfunction, and end stage organ failure. Further studies might provide more information on impact of different antioxidant compounds on the *cysteine-related thiolome* in different organs, plasma and urine, to better address organ-dependent mechanisms and information on off-target effects.

As previously mentioned, *cysteine-related thiolome* profile was changed during HTN development induced by chronic intermittent hypoxia [11], which is resistant to the anti-hypertensive effect of the β-blocker carvedilol [232]. The referred studies are suggestive that *cysteine-related thiolome* might be a valuable precision tool for prediction of HTN development and to response to nutritional or pharmacological interventions.

## 4. Materials and Methods

### 4.1. Animals

Complying with the principles of the 3Rs, this study analyzed stored tissues from healthy animals belonging to different experiments performed in our laboratory. A total of fifty-three male Wistar rats Crl:WI (Han) (Rattus norvegicus L.), with a mean age of 15.6 ± 3.3 weeks, mean body weight at the beginning of the experiments and at sacrifice of 278.1 ± 57.3 g and 343.2 ± 64.1 g respectively, and with an increment in body weight of 66.2 ± 34.6 g were obtained from the NOVA Medical School animal facility. The rats were housed two per cage in polycarbonate cages with wire lids (Tecniplast, Buguggiate, Varese, Italy) and maintained under standard laboratory conditions: artificial 12 h light/dark cycles (9 a.m. to 9 p.m.), at room temperature (22 ± 2.0 °C) and a relative humidity of 60 ± 10%. The rats were maintained on a standard laboratory diet (SDS diets RM1, Special Diets Services, UK) and reverse osmosis water ad libitum. Corncob bedding (Probiológica, Lisbon, Portugal) was used and changed weekly. The animals were specific-pathogen-free, according to FELASA recommendations.

On the last day of the experiment, the rats were anesthetized by intraperitoneal injection with medetomidine (0.5 mg/kg BW; Domitor^®^, Pfizer Animal Health, Auckland, New Zealand) and ketamine (75 mg/kg BW; Imalgene 1000^®^, Mérial, Lyon, France). The animals were decapitated under deep anesthesia, brains were removed from skull, and hippocampus and prefrontal cortex were dissected. The liver and kidneys were also rapidly removed. The right kidney was dissected to obtain the kidney cortex and kidney medulla.

All applicable institutional and governmental regulations concerning the ethical use of animals were followed: the NIH Principles of Laboratory Animal Care (NIH Publication 85-23, revised 1985), the European guidelines for the protection of animals used for scientific purposes (European Union Directive 2010/63/EU), and the Portuguese Law No. 113/2013. All studies in which the animals were involved, were approved by the Institutional Ethics Committee of the NOVA Medical School for animal care and use in research (protocol No. 15/2017/CEFCM).

### 4.2. Quantification of Cysteine-Related Thiolome

The *cysteine-related thiolome* was obtained through the quantification of cysteine, glutathione, and CysGly. These molecules were analyzed in their non-protein-bound forms (LMWT), comprising the reduced (RSH) and the respective disulfides (RSSR) fractions, and in their *S*-thiolated protein (RSSP) forms (CysSSP, GSSP and CysGlySSP, respectively).

Liver (approximately 50 mg), kidney cortex and medulla (approximately 120 mg/each), hippocampus (one) and prefrontal cortex (one) were collected, kept on ice, and immediately homogenized in 400 μL of iced phosphate-buffered saline (PBS 1×) (P4417-100TAB, Sigma-Aldrich, St. Louis, MO, USA). After centrifugation (13,000× *g*, 5 min at 4 °C), an initial volume of 50 μL from the tissue homogenates were used to assess thiol fractions. Thiols were quantified by high performance liquid chromatography (HPLC) with fluorescence detection (HPLC-FD, Shimadzu Scientific Instruments Inc., Columbia, MD, USA) and with the use of standards, as previously described [10,11,233].

The metabolites were separated on a reversed-Phase C18 LiChroCART 250-4 column (LiChrospher 100 RP-18, 5 µm, VWR, Radnor, PA, USA), in column oven at 29 °C on isocratic elution mode for 20 min, at a flow rate of 0.8 mL/min. The mobile phase consisted of a 100 mM acetate buffer (pH 4.5) and methanol (99:1 (*v/v*)). The detection was performed with RF 10AXL fluorescence detector, operating at excitation and emission wavelengths of 385 and 515 nm, respectively.

The protein-bound fraction (RSSP) of each moiety was obtained by subtracting the total free fraction to the total fraction. Moreover, the free oxidized fraction (RSSR) was determined by the difference between free reduced and the total free fractions. In case of concentrations below the limit of quantification, values were represented by the square number of the limit of quantification. The absolute thiols values from tissues were normalized by the weight of the respective tissue and expressed as µM/mg tissue.

### 4.3. Data Analysis

#### 4.3.1. Multivariate Analysis

Multivariate analyses were performed to assess the differences in the *cysteine-related thiolome* among tissues. The dataset consisted of 11 fractions (total, free total, free oxidized, free reduced and protein-bound cysteine; total, free total and protein-bound CysGly; total, free total and protein-bound glutathione) × 153 observations (tissues). Data were mean-centered, pareto-scaled and log transformed before statistical analysis. Principal Component Analysis (PCA) was performed with SIMCA software version 16.0.1 (MKS Umetrics, Umeå, Sweden).

The free reduced and free oxidized glutathione and the free reduced and free oxidized CysGly fractions were mostly near or below the quantification level. As such, they were excluded from the PCA analysis.

#### 4.3.2. Univariate Analysis

Data are presented as the mean ± standard error of the mean (SEM). Metabolites were correlated with age and body weight using Spearman’s correlations, since age, body weight and thiols did not follow the normal distribution. Statistical analysis was performed using GraphPad Prism software version 8 (GraphPad Software, San Diego, CA, USA), and a *p*-value < 0.05 was set to consider statistically significant differences.

## 5. Conclusions

We herein provided original data and an integrated comprehensive review of the literature to support the relevance of cysteine to the kidney and justify its high content on cysteine. Cysteine and its renal metabolism represent a way to control cysteine excess in the body, a source of relevant metabolites for biomass and bioenergetics and a promoter of adaptive responses to stressors. While more studies are needed to clarify the exact contribution of cysteine for kidney function and blood pressure regulation, expanding evidence justifies cysteine metabolic circuits as relevant players beyond its antioxidant activities. Clinical studies on the *cysteine-related thiolome* among individuals in different kidney and related diseases as HTN, as well as on pharmacological and non-pharmacologic interventions are needed to open novel perspectives of this cysteine-related metabolic/redox signature as a valuable tool in precision medicine. 

## Data Availability

The data are contained within the article.
